# Surgeons’ Contributions to Antibiotic Stewardship and Resistance Prevention

**DOI:** 10.1001/jamanetworkopen.2025.21165

**Published:** 2025-07-16

**Authors:** Gabriel Birgand, Nicolas Jacquet, Hubert Johanet, Niki Christou, Patrick Castel, Patrice Baillet, Céline Pulcini

**Affiliations:** 1 Regional Center for Infection Prevention and Control, Region of Pays de la Loire, Nantes University Hospital, France; 2 Cibles et médicaments des infections et de l’immunité, IICiMed, UR 1155, Nantes Université, Nantes, France; 3 Académie Nationale de Chirurgie, Paris, France; 4 National Institute for Health Research Health Protection Research Unit in Healthcare Associated Infections and Antimicrobial Resistance, Imperial College London, London, United Kingdom; 5 Service de chirurgie digestive, générale et endocrinienne, CHU Dupuytren, 2, avenue Martin-Luther-King, 87042 Limoges cedex, France; 6 Sciences Po, CSO, UMR 7116 CNRS, Paris, France; 7 Université de Lorraine, INSPIIRE, Nancy, France; Université de Lorraine, CHRU-Nancy, Centre Régional en Antibiothérapie du Grand Est AntibioEst, Nancy, France

## Abstract

This survey study evaluates how surgeons in France understand their role in preventing antibiotic resistance.

## Introduction

The risk of postoperative infections is mitigated through a series of preventive and curative measures.^1^ While the capacity of health care systems to deliver safe surgery relies on the availability of effective antibiotics, the inappropriate use of these drugs—both for prophylaxis and therapy—remains a significant concern, as it contributes to the emergence and spread of antibiotic resistance (ABR), ultimately threatening progress in surgical care.^2,3^ Although lack of clarity regarding the ownership of the antibiotic prescription has been previously noted, the specific role of surgeons in prescribing remains underexplored.^4^ We conducted an nationwide, exploratory, anonymous, self-administered online questionnaire to evaluate the perceptions, roles, and responsibilities of surgeons regarding ABR.

## Methods

The questionnaire for this survey study was disseminated between May 13 and October 10, 2024, through the French National Academy of Surgery and national professional societies to surgeons working in secondary and tertiary care. Data were gathered from respondents about their perception of the impact of ABR in surgery in general and in routine practice, roles and responsibilities in prescribing and administrating surgical antibiotic prophylaxis (SAP), and involvement in the diagnosis and treatment of postoperative infections (eAppendix in Supplement 1). Univariable analyses were performed using a 2-tailed Fisher exact test or Wilcoxon test. Statistical analyses were performed with Stata version 18 (StataCorp), with *P* < .05 considered statistically significant. We obtained ethical review from the French Society of Infectious Diseases to conduct this study. All participants received an information letter, and consent was obtained before their interviews. The reporting adheres to AAPOR Best Practices for Survey Research.

## Results

The survey was completed by 234 surgeons (32.2% female and 55.2% with >20 years’ experience), with 48 (20.5%) orthopedic surgeons , 62 (26.5%) digestive surgeons, 101 (43.2%) gynecological surgeons, and the remaining from other surgical specialties. Regarding the perception of ABR in surgery, 114 of 234 respondents (48.7%) agreed that ABR would threaten the ability to provide quality surgical care in the near future in France (Table 1). Among 221 respondents, 53 (23.9%) encountered one and 34 (15.4%) several issues with ABR during the month prior to the survey that complicated the surgical management of their patients. Among 129 surgeons not affected by ABR, 124 (96.1%) thought this could be the case in the coming 10 years, first for infection treatment (112 [90.3%]) and second for SAP (57 [45.9%]).

**Table 1.** Reported Differences by Surgical Specialties in the Perceptions and Roles of Surgeons Regarding Antibiotic Resistance

Question	No./total No. (%)				*P* value
	Total	Orthopedic	Digestive	Gynecological	
**Perception of the impact of antibiotic resistance in surgery**						
In your opinion, does antibiotic resistance threaten the ability to provide quality surgical care in the near future in France?					
Agree or fully agree	114/234 (48.7)	29/48 (60.4)	31/62 (53.0)	44/101 (49.6)	.26
Disagree or fully disagree	45/234 (19.2)	8/48 (16.7)	5/62 (8.1)	25/101 (24.7)	.03
In the past month, did you have issues with antibiotic resistance that complicated the surgical management of your patients?					
Never	134/221 (60.6)	26/44 (59.1)	29/60 (48.3)	69/96 (71.9)	.01
But it could happen in the next 10 years	124/129 (96.1)	25/26 (96.1)	27/28 (96.1)	63/66 (95.4)	.93
Regarding surgical indications	16/124 (12.9)	3/25 (12.0)	4/27 (14.8)	8/63 (12.7)	.99
Regarding SAP practices	57/124 (45.9)	12/25 (48.0)	13/27 (48.1)	29/63 (46.0)	.88
Regarding antibiotic prescribing to treat infection	112/124 (90.3)	23/25 (92.0)	25/27 (92.6)	55/63 (87.3)	.60
Once	53/221 (23.9)	9/44 (20.4)	23/60 (38.3)	18/96 (18.7)	.02
Several times	34/221 (15.4)	9/44 (20.4)	8/60 (13.3)	9/96 (9.4)	.007
Regarding surgical indications	4/34 (11.8)	3/9 (33.3)	0/8	0/9	.09
Regarding SAP practices	7/34 (20.6)	2/9 (22.2)	2/8 (25.0)	2/9 (22.2)	.93
Regarding antibiotic prescribing to treat infection	32/34 (94.1)	9/9 (100)	7/8 (87.5)	8/9 (88.9)	.54
Do you consider yourself sufficiently informed about the phenomenon of antibiotic resistance and its evolution?					
Well or very well informed	73/230 (31.7)	19/48 (39.6)	17/61 (27.9)	23/98 (23.5)	.003
Poorly or not informed	77/230 (33.5)	11/48 (22.9)	25/61 (40.9)	36/98 (36.7)	.12
Do you consider yourself sufficiently informed about the measures to control antibiotic resistance in your surgical practice?					
Well or very well informed	73/231 (31.6)	20/48 (41.7)	16/61 (26.2)	21/99 (21.2)	.003
Poorly or not informed	94/231 (40.7)	14/48 (29.2)	25/61 (40.9)	50/99 (50.5)	.02
**Roles and responsibilities regarding infection related care**						
Are the roles of the different actors clearly defined for the medical management of infections in your department/practice?					
Not at all	17/211 (8.1)	0/46	6/52 (11.5)	10/92 (10.9)	.10
A little	44/211 (20.8)	3/46 (6.5)	15/52 (28.8)	24/92 (26.1)	.01
Largely	80/211 (37.9)	18/46 (39.1)	18/52 (34.6)	33/92 (35.9)	.51
Totally	70/211 (33.2)	25/46 (54.3)	13/52 (25.0)	25/92 (27.2)	.006
Do you extend SAP during the postoperative phase to secure patient care?					
Never	107/227 (47.7)	37/48 (72.1)	23/59 (37.9)	36/95 (33.9)	<.001
Sometimes	106/224 (47.3)	10/48 (20.8)	34/59 (57.6)	55/95 (57.9)	<.001
Often	11/224 (4.9)	1/48 (2.1)	2/59 (3.4)	4/95 (4.2)	.02
Always	0/224	0/48	0/59	0/95	-
**Frequency of surgeon’s involvement in the management of patients regarding**						
Selecting SAP at the beginning of the surgery					
Never	27/163 (16.6)	9/41 (21.9)	7/41 (17.1)	11/65 (16.9)	.26
Sometime	63/226 (27.9)	7/48 (14.6)	19/60 (31.7)	31/96 (32.3)	.13
Often	61/226 (26.9)	11/48 (22.9)	17/60 (28.3)	29/96 (30.2)	.61
Always	75/226 (33.2)	21/48 (43.7)	17/60 (28.3)	25/96 (26.0)	.02
Diagnosis of suspected postoperative infections					
Never	2/218 (0.9)	1/48 (2.1)	0/58	1/92 (1.1)	.69
Sometime	8/226 (3.5)	0/48	2/60 (3.3)	4/96 (4.2)	.28
Often	65/226 (28.8)	4/48 (8.3)	18/60 (30.0)	39/96 (40.6)	.001
Always	151/226 (66.8)	43/48 (89.6)	40/60 (66.7)	52/90 (54.2)	<.001
Antibiotic prescribing to treat postoperative infection					
Never	4/203 (1.9)	4/37 (10.8)	1/57	0/91	<.001
Sometime	21/224 (9.4)	10/47 (21.3)	3/60 (5.0)	5/96 (5.2)	.008
Often	88/224 (39.3)	12/47 (25.5)	23/60 (38.3)	47/96 (48.9)	.04
Always	111/224 (49.5)	21/47 (44.7)	34/60 (56.7)	44/96 (45.8)	.44
Clinical follow-up of patients treated for postoperative infection					
Never	3/213 (1.4)	2/46 (4.3)	1/56 (0.2)	1/93 (1.1)	.26
Sometime	12/226 (5.3)	2/48 (4.2)	4/60 (6.7)	3/96 (3.1)	.23
Often	70/226 (30.9)	10/48 (20.8)	19/60 (31.7)	36/96 (37.5)	.18
Always	141/226 (62.4)	34/48 (70.8)	37/60 (61.7)	56/96 (58.3)	.54

Abbreviation: SAP, surgical antibiotic prophylaxis.

The role of the different actors in the medical management of infections in their surgical department was considered clearly and fully defined by 70 of 211 respondents (33.2%), with a difference between orthopedic and other specialties (orthopedic: 25 of 46 [54.3%]; digestive: 13 of 52 [25.0%]; gynecological: 25 of 92 [27.2%]). The selection of SAP at the beginning of the surgery always involved surgeons in 33.2% of cases (75 of 226) or often in 26.9% (61 of 226). Almost half of surgeons (106 of 224 [47.3%]) declared sometimes and 11 (4.9%) often extending SAP during the postoperative phase to secure patient care. This practice was more commonly associated with younger male surgeons (Table 2). Most (151 of 226 [66.8%]) were systematically involved in the diagnosis of suspected postoperative infections, and 111 (49.5%) in the antibiotic prescribing process to treat them. Senior surgeons were involved in first-line treatment of infections (69.0% a lot or exclusively) followed by the infectious diseases team (63.2%), junior surgeons (43.7%), and anesthesiologists (35.3%). This pattern was more commonly observed among younger female surgeons working in public facilities (Table 2).

**Table 2.** Reported Differences by Surgeon Characteristics in the Perceptions and Roles of Surgeons Regarding Antibiotic Resistance

Characteristic	Total, No./total No. (%)	Agree or fully agree that antibiotic resistance threatens the ability to provide quality surgical care in the near future in France			Well or very well informed about measures to control antibiotic resistance in surgical practice			Never extend surgical antibiotic prophylaxis during the postoperative phase to secure patient care			Often or always involved in antibiotic prescribing to treat postoperative infection		
		No./total No. (%)	OR (95% CI)	*P* value	No./total No. (%)	OR (95% CI)	*P* value	No./total No. (%)	OR (95% CI)	*P* value	No./total No. (%)	OR (95% CI)	*P* value
Gender, female	73/231 (31.6)	34/113 (30.1)	0.87 (0.19-1.52)	.63	16/70 (22.9)	0.53 (0.27-1.01)	.04	27/106 (25.5)	0.57 (0.32-1.02)	.05	65/196 (33.2)	1.98 (0.71-5.56)	.18
Age category													
20-49 y	55/227 (24.2)	27/111 (24.3)	1 [Reference]	NA	6/68 (8.8)	1 [Reference]	NA	33/104 (31.7)	1 [Reference]	NA	50/194 (25.8)	1 [Reference]	NA
50-79 y	172/227 (75.8)	84/111 (75.7)	0.99 (0.54-1.82)	.97	62/68 (91.2)	4.73 (1.86-12.03)	<.001	71/104 (68.3)	0.46 (0.24-0.88)	.01	144/194 (74.2)	0.43 (0.12-1.52)	.18
Type of facility for primary work sector^a^													
Public	120/233 (51.5)	57/113 (50.4)	1 [Reference]	NA	38/72 (52.8)	1 [Reference]	NA	52/107 (48.6)	1 [Reference]	NA	103/199 (51.8)	1 [Reference]	NA
Private	113/233 (48.5)	56/113 (49.6)	1.08 (0.64-1.81)	.75	34/72 (47.2)	0.94 (0.64-1.64)	.83	55/107 (51.4)	1.27 (0.75-2.16)	.36	96/199 (48.2)	0.73 (0.31-1.69)	.46
Experience after the internship													
0-20 y	102/232 (44.0)	51/113 (45.1)	1 [Reference]	NA	19/71 (26.8)	1 [Reference]	NA	46/105 (43.8)	1 [Reference]	NA	90/198 (45.5)	1 [Reference]	NA
>20 y	130/232 (56.0)	62/113 (54.9)	0.91 (0.54-1.53)	.73	52/71 (73.2)	3.03 (1.61-5.69)	<.001	59/105 (56.2)	0.99 (0.58-1.69)	.97	108/198 (54.5)	0.49 (0.19-1.25)	.13

Abbreviations: NA, not applicable; OR, odds ratio.

^a^ One participant can work in different types of facilities.

## Discussion

This survey found that surgeons were aware of the consequences of ABR on the effectiveness of antibiotics for treatment. Paradoxically, half of them extended the duration of SAP postoperatively. This exploratory survey provides some insights to develop interventions to improve surgeons’ knowledge of ABR and clarify roles and optimize infection management in surgical patients.^5^ Particular attention should be paid to surgical specialties generating a large burden of infections requiring antibiotic use.^6^ The study’s findings are limited by the restriction to surgeons working in France, impairing the generalizability; the self-reported design; and not addressing prescribing behaviors or outcomes.

## Notes

Supplement 1. **eAppendix.** Questionnaire Used for Survey

Supplement 2.
Data Sharing Statement
