# Supplementary material for: Surgeons’ Contributions to Antibiotic Stewardship and Resistance Prevention
Source: JAMA Netw Open. 2025 Jul 16;8(7):e2521165. doi: 10.1001/jamanetworkopen.2025.21165 (PMC12268483; doi:10.1001/jamanetworkopen.2025.21165)
Supplement: Supplement 1. — eAppendix. Questionnaire Used for Survey [file jamanetwopen-e2521165-s001.pdf]

Supplemental Online Content

Birgand G, Jacquet N, Johanet H, et al. Surgeons' contributions to antibiotic stewardship and resistance prevention. *JAMA Netw Open.* 2025;8(7):e2521165. doi:10.1001/jamanetworkopen.2025.21165

eAppendix. Questionnaire Used for Survey

This supplemental material has been provided by the authors to give readers additional information about their work.

© 2025 Birgand G et al. *JAMA Network Open*.

eAppendix. Questionnaire Used for Survey

General information

- How old are you?

Answers: 20 to 29 years/ 30 to 39 years/ 40 to 49 years/ 50 to 59 years/ 60 to 69 years/ 70 to 79 years

- What is your gender?
- What position do you hold?

Answers: Senior surgeon/Intern in surgery/Medical student

- What is your surgical specialty?

Answers: Gynaecology Surgery/ Digestive Surgery/ Orthopaedic surgery/ Urology / Neurosurgery / Cardiac Surgery/ Paediatric Surgery/ Vascular surgery/ Maxillo-facial surgery/ Other

- In which country do you mainly practice?
- In what type(s) of facility(s) do you primary work?

Answers: Regional or university hospital/ Other public hospital/ Non for profit private hospital/ For profit private hospital/ Other

Perception of the impact of antibiotic resistance in surgery

- In your opinion, does antibiotic resistance threaten the ability to provide quality surgical care in the near future in France?

Quotation from 1 – totally disagree to 5 – Fully agree NA

- Over the past month, have you been confronted with antibiotic resistance problems that have complicated the surgical care of your patients?

Answers: Never/Once/several times/ NA

- Do you consider yourself sufficiently informed about the phenomenon of antibiotic resistance and its evolution?

Quotation from 1 – Not informed at all to 5 – Very well informed NA

- Do you consider yourself sufficiently informed about measures to control antibiotic resistance in your surgical practice?

Quotation from 1 – Not informed at all to 5 – Very well informed NA

Roles and responsabilities

- Are the roles of the different actors clearly defined for the medical management of infections in your department/practice?

Quotation from 1 – very poorly to 5 – Very well NA

- Which professionals are responsible for the medical management of infections (suspected or proven) in your department/practice?

Answers: Not at all/A bit/A lot/Exclusively/NA, applied to the following categories: Surgeons/Anesthetists/Intern in Surgery/Infectious diseases physician/Medical doctor with dedicated time on the surgical ward/ Other professional

- Regarding the management of infections and infectious risks, how would you rate the quality of your collaboration with:

© 2025 Birgand G et al. *JAMA Network Open*.

Quotation from 1 – very bad to 5 – Very good NA, applied to: Operating nurses/ Anesthesia team (Anesthetist, Anesthesy nurse)/Infection control team/ Infectious diseases team/Others

- Do you find yourself prolonging antibiotic prophylaxis during the post-operative phase to secure the care of your patients?

Answers: Never/ Sometime/ Often/ Always

- Indicate your frequency of involvement in the care of your patients regarding:
  - The choice of surgical antibiotic prophylaxis

Answers: Never/ Sometime/ Often/ Always

  - The diagnosis of post-operative infections

Answers: Never/ Sometime/ Often/ Always

  - The prescription of antibiotic treatment for a suspected post-operative infection

Answers: Never/ Sometime/ Often/ Always

  - The clinical follow-up of the patient treated for a suspected post-operative infection and the re-evaluation of antibiotic therapy:

Answers: Never/ Sometime/ Often/ Always

© 2025 Birgand G et al. *JAMA Network Open*.
