# Supplementary material for: Surgeons’ Contributions to Antibiotic Stewardship and Resistance Prevention
Source: JAMA Netw Open. 2025 Jul 16;8(7):e2521165. doi: 10.1001/jamanetworkopen.2025.21165 (PMC12268483; doi:10.1001/jamanetworkopen.2025.21165)
Supplement: Supplement 2. — Data Sharing Statement [file jamanetwopen-e2521165-s002.pdf]

Data Sharing Statement

Birgand. Surgeons’ Contributions to Antibiotic Stewardship and Resistance Prevention. *JAMA Netw Open*. Published July 16, 2025. doi:10.1001/jamanetworkopen.2025.21165

Data
Data available: Yes
Data types: Deidentified participant data
How to access data: gabriel.birgand@chu-nantes.fr
When available: With publication

Supporting Documents
Document types: Statistical/analytic code
How to access documents: gabriel.birgand@chu-nantes.fr
When available: With publication

Additional Information
Who can access the data: researchers whose proposed use of the data has been approved
Types of analyses: for a specified purpose
Mechanisms of data availability: after approval of a proposal
